# Artificial Intelligence-Empowered 3D and 4D Printing Technologies toward Smarter Biomedical Materials and Approaches

**DOI:** 10.3390/polym14142794

**Published:** 2022-07-08

**Authors:** Raffaele Pugliese, Stefano Regondi

**Affiliations:** NeMO Lab, ASST GOM Niguarda Cà Granda Hospital, 20162 Milan, Italy; stefano.regondi@nemolab.it

**Keywords:** additive manufacturing, artificial intelligence, 4D printing, 5D printing, smart materials, functional materials, biomedicine, open-loop AI, closed-loop AI

## Abstract

In the last decades, 3D printing has played a crucial role as an innovative technology for tissue and organ fabrication, patient-specific orthoses, drug delivery, and surgical planning. However, biomedical materials used for 3D printing are usually static and unable to dynamically respond or transform within the internal environment of the body. These materials are fabricated ex situ, which involves first printing on a planar substrate and then deploying it to the target surface, thus resulting in a possible mismatch between the printed part and the target surfaces. The emergence of 4D printing addresses some of these drawbacks, opening an attractive path for the biomedical sector. By preprogramming smart materials, 4D printing is able to manufacture structures that dynamically respond to external stimuli. Despite these potentials, 4D printed dynamic materials are still in their infancy of development. The rise of artificial intelligence (AI) could push these technologies forward enlarging their applicability, boosting the design space of smart materials by selecting promising ones with desired architectures, properties, and functions, reducing the time to manufacturing, and allowing the in situ printing directly on target surfaces achieving high-fidelity of human body micro-structures. In this review, an overview of 4D printing as a fascinating tool for designing advanced smart materials is provided. Then will be discussed the recent progress in AI-empowered 3D and 4D printing with open-loop and closed-loop methods, in particular regarding shape-morphing 4D-responsive materials, printing on moving targets, and surgical robots for in situ printing. Lastly, an outlook on 5D printing is given as an advanced future technique, in which AI will assume the role of the fifth dimension to empower the effectiveness of 3D and 4D printing for developing intelligent systems in the biomedical sector and beyond.

## 1. Introduction

Smart materials, also called intelligent or responsive materials, are designed materials that have the ability to dynamically respond to external stimuli, adapting their features and functions for a specific need of applications [1,2,3,4,5,6]. Usually, such materials respond to stimuli such as temperature, pH, humidity, light, electromagnetism, ion concentration, and mechanical force [7,8,9,10]. In addition, they are able to perform living-like functions such as self-healing, self-assembly, shape memory, self-evolving, sensing, and actuating [11,12,13,14].

The design and experimentation of smart structures has pushed researchers to develop different strategies in terms of behavior enhancement and property in relationship to specific applications [15,16]. However, smart materials have become increasingly complex (in terms of structures, nano- and micro-topology, physical-chemical and mechanical features), outpacing traditional manufacturing due to the intrinsic mechanical and control limitations of these machines [17].

In recent years, additive manufacturing (AM), also called 3D printing, has emerged as a versatile technique and a valuable alternative to traditional manufacturing for the fabrication of complex materials through a layer-by-layer approach, thus resulting in new types of biomedical equipment, scaffolds, wearable devices, soft robotics, actuators, and flexible electronics [18,19,20,21,22,23,24,25,26,27].

**Figure 1:**
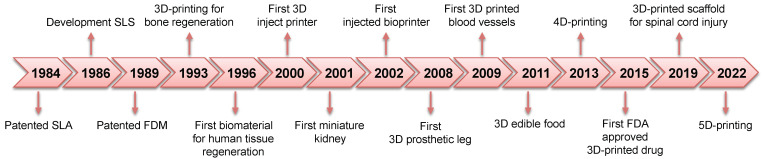
Additive manufacturing history and milestones in the biomedical field.

In the biomedical sector, 3D printing has played a crucial role as an innovative technology for tissue engineering, organ fabrication, regenerative medicine, and drug delivery [28] (Figure 1). Furthermore, 3D printing has attracted considerable interest in this field because it allows the development of patient-specific personalized orthoses, prostheses, craniofacial implants, and medical devices according to personal data [29,30,31,32,33,34,35,36]. To be emphasized, 3D printing has a significant impact on medical education and surgical planning as 3D anatomical models can be printed by perfectly reproducing the microscopic anatomical structures of tissues and organs, thus having a positive impact in terms of time required, efficiency, accuracy, and success of the surgery, but can also be used for training new surgeons [37,38,39].

Currently, the methods of 3D printing used in biomedical fields mainly include the following: fused deposition modeling (FDM), selective laser sintering (SLS), stereolithography (SLA), and direct-ink-writing (DIW) [40,41,42,43,44]. Based on the specific needs, with such methods, these devices or implants can be fabricated in various materials, from polymers to hydrogels, up to metal alloys [40,45]. In addition, through 3D printing, they are fabricated with different designed topologies with an extremely reduced amount of time and cost. Other benefits of 3D printing are the ease of customizing designs, the possibility of printing complex shapes in a controlled manner, and the ability to create nature-resembling structures to improve performance to satisfy customers’ needs within a short turnaround time [46]. All these features are not measured and are precisely controlled in traditional manufacturing.

However, although 3D-printed structures successfully mimic complex anatomical structures from a geometrical-topological point of view, most 3D-printed materials are static and are unable to spontaneously change or adapt their features in response to the surrounding dynamic environment. 

A step forward was given by 4D printing, proposed for the first time by Skylar Tibbits [47] as 3D printing coupled with transformation capabilities (i.e., shape/color changing, or structure healing) over time [48,49]. Hence, the additional dimension compared to 3D printing, namely, the fourth dimension, has been defined as “time”, since it is directly connected to the change of shape, properties, and functionality of the printed material over time following its exposure to physical-chemical stimuli [50].

Notably, using 4D printing materials enables dynamic properties to open an attractive path for biomedical and tissue engineering applications [51]. For instance, Hendrikson et al. [52] reported the use of polyurethane to print the 4D-shape memory polymers with controllable time-dependent shape changes that mechanically stimulate the cell’s morphological functionality. During 4D-scaffold deformation, cells seeded on the scaffold are elongated by mechanical stimulation, allowing them to be implanted into patients using minimally invasive surgery. A different approach has been given by Malachowski et al. [53], which developed a thermally responsive theragripper composed of biodegradable poly(propylene-fumarate) and biocompatible poly(N-isopropylacrylamide-co-acrylic acid) for the controlled release of the multi-fingered drug through its layers and pores. The theragripper is closed at temperatures greater than 32 °C, which allows it to spontaneously grasp the tissue as it enters the body from a cold state and subsequently to be effectively immobilized in a specific site, allowing the prolonged and controlled release of the drug. This strategy could be useful for patients with inflammatory bowel disease or gastrointestinal cancers since it could avoid the systemic medications of chemotherapy, thus reducing the dosage and the related side effects. The 4D printing may also be used for developing shape memory thermosets endoluminal devices (i.e., tracheal stents), as recently reported by Zarek et al. [54].

Despite these potentials, 4D printed dynamic devices are still in their infancy of development. The following challenge lies in the design of materials that are both dynamic and biocompatible after printing: extremely important factors to be implemented in the medical field, where organs and tissues are by definition complex and dynamic multifunctional environments. Another major drawback lies in the nature of 3D- and 4D printing where designed structures are typically manufactured on a planar and flattened substrate (namely, ex situ printing), and then transferred to the target non-planar surfaces, such as those of the human body. Therefore, the printing procedure is fully deterministic, with limited “real-time knowledge” of the target geometry—except through computed tomography or laser scanning—thus leading to a possible mismatch between the printed part and target surfaces. 

These issues represent key challenges that need to be addressed in the coming years to foster the spread of AM technologies. One way to get around them could be the use of artificial intelligence (AI): a tool that allows training machines for the development of human-like capabilities in order to predict and represent the statistically significant and most likely behavior of a phenomenon [55,56]. AI and its subset machine learning (ML) can be a powerful tool to enlarge the applicability of 3D- and 4D printing, reduce the time of manufacturing, and boost the design space.

**Figure 2:**
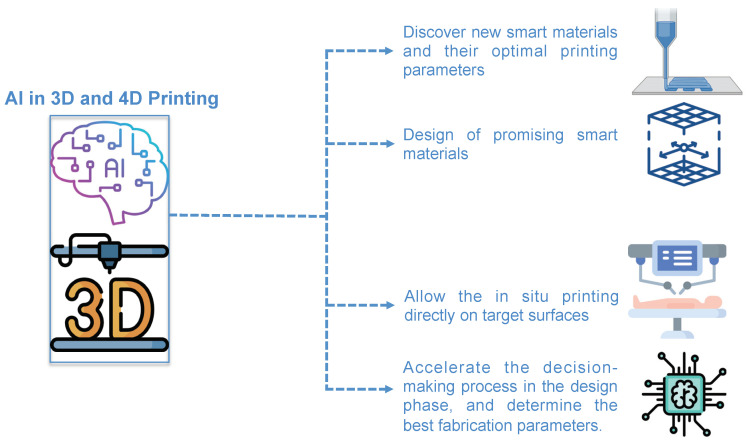
The possible uses of AI in 3D- and 4D printing applications. This figure was designed using icon made by freepik and berkhicon from flaticon.

Indeed, the use of optimized ML and AI algorithms in 3D- and 4D printing are expected to perform the following: (1) discover new smart materials and their optimal printing parameters; (2) accelerate the smart material design by selecting promising ones with desired architectures, properties, and functions (avoiding the lengthy trial-and-error production phase); (3) allow the in situ printing directly on target surfaces achieving high-fidelity of microstructures compared to the ex situ printing; (4) predict and identify relationships between specific materials and process setups that have not yet been tried. Besides, AI and ML algorithms can be incorporated into the AM framework at multiple levels, such as to accelerate the decision-making process in the design phase, determine the best fabrication parameters, identify the ideal printability orientation, and decrease process time [57] (Figure 2).

In this review, we provide an overview of 4D printing as a fascinating tool for designing advanced smart materials. We then discuss the recent progress in AI-empowered 3D- and 4D printing with open-loop and closed-loop methods, in particular regarding the field of smart materials design for biomedical approaches.

Furthermore, as recently reported by Milazzo and Libonati [58], we give an outlook on the “5D printing” technique, in which AI and ML will assume the role of the fifth dimension to empower the effectiveness of AM in biomedical approaches in real-time. Lastly, we will briefly discuss the regulatory standpoint for managing AI technologies.

## 2. 4D Printing

From its first appearance in 2013, 4D printing demonstrated a radical shift in AM [47,59,60]. Tibbits defined 4D printing as multi-material printing with the capability to transform over time or a customized material system that can change shape, structure, or function directly off the print bed [47]. The fourth dimension was described with the formula of “3D printing + time”, emphasizing that printed structures are no longer simply static or dead objects, but rather they are active and dynamic structures that can spontaneously transform. 

**Figure 3:**
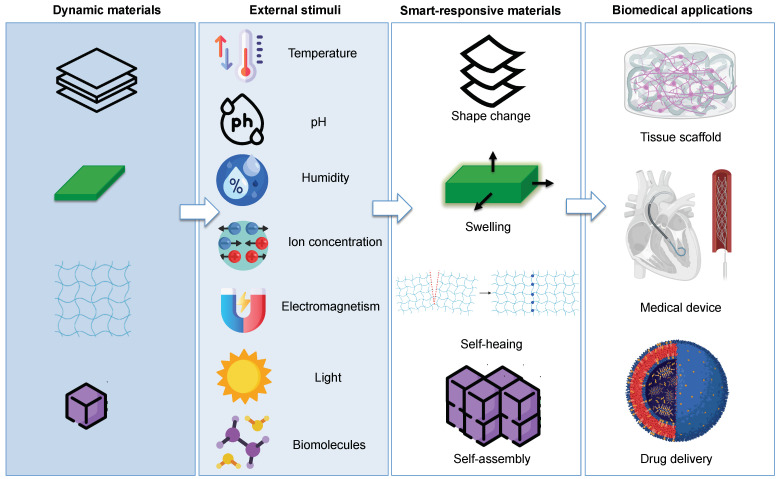
Schematic representation of different types of stimuli, and responses observed in smart materials in terms of shape-shifting, swelling, self-assembly, self-repair, and their possible use in biomedical applications.

With the evolution of this technology, the concept of 4D printing has been expanded by incorporating the product design into a flexible and intelligent material based on 3D printing [61]. Therefore, the structures can deform, swell, self-assemble, or self-repair according to a pre-designed path under specific conditions of time and upon exposure to external stimuli. Such stimuli that are strictly connected with the changes in shape, properties, and functionality of 4D printed structures can be both physical (temperature, humidity, light, electromagnetism, and mechanical force) and chemical (pH, chemical reactions, ion concentration, cross-linking, redox state of metal ions) and can be applied sequentially or simultaneously to trigger a permanent or temporary change in the 4D printed objects (Figure 3). In addition, such stimuli can also be of a biological nature (e.g., biomolecules, enzymes, and cell traction force), which are of particular interest for the fabrication of 4D-bioprinted engineered living scaffolds that allow tissue repair and regeneration or a replicating cell population of living organisms [62,63,64].

Hence, 4D printing represents a glimpse into the world of smart materials that can respond or adapt to environmental changes, biometric information, body temperature, pressure, or sweat, to name a few. 

Therefore, it is clear that the stimuli-responsive materials must possess the following two key features to be used in 4D printing: (1) printability according to the guidelines of AM technologies and (2) sensitivity to a stimulus, achievable intrinsically from the polymer matrix or by incorporating additives or fillers into the polymer matrix [7,8].

Below, we elucidate some key aspects that distinguish 4D printing (self-repair, self-adaptability, shape-shifting, and self-assembly) as useful for creating the above-mentioned dynamic and controlled environments that are not exclusive to the biomedical field.

### 2.1. Self-Adaptability

Self-adaptive structures are fascinating applications of 4D printing. Through 4D printing, self-organizing structures can be obtained using materials that mimic DNA strands with complementarity sequences that couple under appropriate physical conditions. By doing so, the building blocks of specially programmable biomaterials can be induced to self-organize on multiple length scales to recapitulate the desired tissue architecture or to precisely control the composition and spatial distribution of cells in manufactured tissues that must mimic those natives [65]. 

A prominent example of self-adaptive material has been reported by Zarek et al. [54]. The authors fabricated 4D printed, customizable endoluminal cylindrical stents via SLA using methacrylated polycaprolactone (PCL-MA) as the stimuli-responsive material. This PCL-MA-based stent transits from a temporary closed state at room temperature (i.e., 20 °C) into a permanently open state at body temperature (i.e., 37 °C), enabling a minimally invasive insertion and better fitting of the stent at the damage site without the need for surgical traction.

In addition, using 4D printing, it is possible to embody self-sensing or self-actuation directly into a material so that external electromechanical systems are not necessary [47], thus decreasing the number of printing parts, assembly time, material and energy costs, which is extremely useful for electromedical and electromechanical systems.

### 2.2. Self-Repair

The self-repair or error-correction capability is another key feature of 4D printing. As reported by Taylor et al. [66], self-repair is defined as the property that enables a material to intrinsically and automatically heal damage, restoring itself to normality. Therefore, these materials are able to repair the damage themselves and regain the associated mechanical properties without human intervention or an external stimulus to promote the initiation or extent of self-repair but rather due to the molecular diffusion of ionic cross-links among the 4D printed polymer matrix. The self-repair requires rebonding a material to its original shape or condition, for example, by cutting a gel in half and then allowing it to bond back together [67]. 

Indeed, hydrogels are the most promising materials for self-healing due to their tunable physical and chemical properties [68]. In the case of self-healing hydrogels, non-covalent interactions (such as an ionic bond, hydrogen bond, hydrophobic interaction, Van der Waals interactions, electrostatic attractions, and ππ stacking) are generally utilized, separately or in combination, to self-mend damage or to restore their original properties [69,70]. The 4D printed self-healing hydrogels show enormous advantages as they not only have the ability to extend their half-life but also lead to an increase in the durability, reliability, reusability of the material and, in some specific applications such as wound dressings, contact lenses, scaffolding for meniscus or cartilage, increase safety by avoiding sagging caused by the accumulation of cracks or breaks [69].

### 2.3. Shape-Shifting

**Figure 4:**
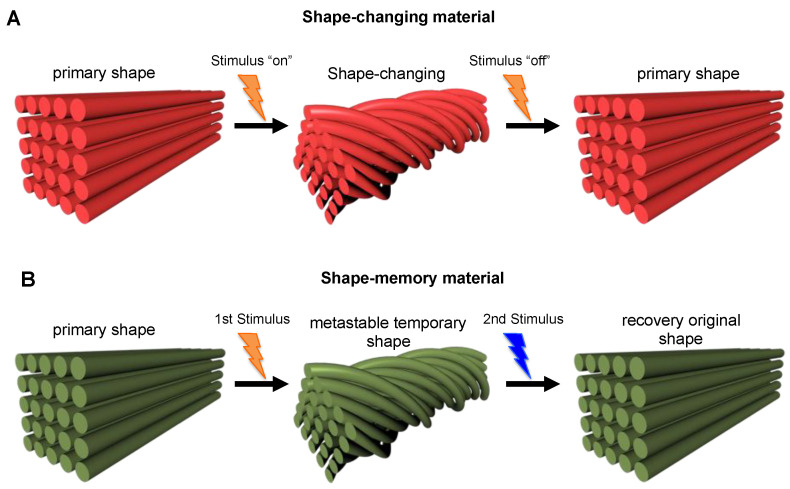
Illustration of (**A**) a shape-changing material with the “on-off” mechanism, and of (**B**) a shape-memory material with the two programming steps in which the structure of the material is deformed from its primary shape following the application of a stimulus and then kept in a temporary metastable shape until a second stimulus is applied allowing the recovery of the original shape.

The shape-shifting materials can take and hold any possible shape, or folding, bending, or twisting following applied stimuli, thus paving the way for a new type of multifunctional material that could be used in a wide range of applications, from medicine and biotechnology to robotics. As reported by Momeni et al. [49] and Zhou et al. [51], the shape-shifting materials could be divided into the following two categories: shape-changing materials and shape-memory materials. A shape-changing material changes its shape immediately upon the application of a stimulus and returns to its original shape immediately after the stimulus has been removed (Figure 4A). Therefore, this type of material works with an “on-off” mechanism, which is usually limited to changes in expansion swelling, twisting, or volume shrinkage [51]. Instead, the shape-memory behavior involves a two-step process (Figure 4B). In step one, the material is deformed from its primary shape following the application of a stimulus, thus reaching a temporary metastable shape, which is maintained until a second appropriate stimulus (which may be different from the previous one) is applied to allow the material to recover its original shape (step 2). Therefore, such shape-memory materials possess the capability to “memorize” and maintain a temporary shape until an appropriate stimulus is applied, while shape-changing materials cannot, thus immediately return to their original conformation as soon as the stimulus is removed. 

The difference between shape-changing and shape-memory may seem nuanced, but in reality, it has important implications for biomedical applications. For instance, with a similar approach to that reported by Zarek et al. [54] for the 4D-printed self-adaptive endoluminal tracheal stent, Wan et al. exploited the shape-changing properties of poly(D,L-lactide-co-trimethylene carbonate) to 4D-print shape-changing patient-specific flower-shaped intravascular stents via DIW [71]. The authors showed that the stents could rapidly self-expand from a closed deformed shape when warmed to 37 °C and return to their original shape when they are cooled (so when the warm temperature stimulus is removed). Instead, Kim et al. fabricated a kirigami-inspired 4D-printed polyurethane-based bifurcated stent using FDM [72]. This bifurcated stent possesses shape-memory properties; when heated to its glass temperature of 55 °C, the stent deforms from its open “Y”-shaped configuration to an “I”-shaped temporary metastable closed configuration, in which the branching of the tube bends into a single tube of smaller diameter. The 4D-printed stent, in the temporary metastable configuration, can thus travel through the main vessel and, upon reaching the bifurcation of the target vessel, the original “Y”-shaped configuration can be recovered by applying a second specific stimulus, which is to increase the temperature to 60 °C. In this way, using this innovative shape-memory method, it will be possible to easily implant a stent at the bifurcation of the target site (even if it is difficult to reach) because, thanks to its temporary metastable configuration, it will be able to pass through the main vessels in a minimally invasive way. 

### 2.4. Self-Assembly

The concept of self-assembly is not new and it is increasingly used in many application fields such as nanomedicine, biotechnology, architecture, infrastructure and other industry scenarios [73]. By definition, self-assembly is the process in which the components of a system, be they atoms, molecules, particles, or polymers, organize themselves autonomously and with free energy into ordered and/or functional structures as a consequence of specific interactions or stimuli [74]. 

Self-assembly processes are ubiquitous in nature (e.g., minerals, shells, pearls, corals, bones, teeth, wood, silk). Indeed, as reported by Shuguang Zhang [75], nature has found a fascinating way of using the self-assembly phenomenon, allowing molecules or structures to organize themselves hierarchically from the nano- to the mesoscale level, thus leading to exceptional properties. Just think of the formation of complex biological machines such as ribosomes, ATP synthase, membrane channels, and hemoglobin.

In 4D printing, the concept of self-assembly is very attractive not only for the fabrication of responsive tissue engineering scaffolds to mimic the complex structure of the extracellular matrix (ECM) of damaged tissues [76], but also for the transfer of parts of equipment within the human body [77]. Certainly, the future development of 4D printing will focus on a variety of self-assembly capabilities and properties of free energy that must be functionally incorporated into the material for developing single parts that can be 4D printed with small printers and then self-assembled into larger structures, such as space antennas, satellites, or international space stations as envisaged by Tibbits and colleagues [78]. Further attractive applications of 4D-self-assembly include self-assembling buildings, especially in non-industrialized zones or war zones where elements can come together to produce a finished building with minimal human involvement [79], and reconfigurable robotic systems with different degrees of freedom (DOFs) [80] in order to serve different and complex mechanical (i.e., locomotion), and/or “programming matter” that encodes structural and functional information of biological-inspired assembly systems [81].

## 3. Open-Loop AI for 3D Printing

Although 4D printing has exerted a positive impact on different biomedical fields and beyond, many limitations and challenges remain to be overcome. Surely, the challenges and prospects for the progress of 4D printing technology lie in the ability to design and integrate chemistry, form and function in materials in order to allow dynamic and complex actions such as self-adaptability, self-repair, shape-changing, shape-memory, and self-assembly, so far not always easy to make. 

In our opinion, it is in this context that the rise of AI could propel these technologies forward by expanding their applicability, increasing the design space for smart materials by selecting promising ones with desired architectures, properties and functions, and reducing production times.

For instance, open-loop AI-based 3D printing leverages the acquisition of information on the geometry of the target surface before the manufacturing process (Table 1 summarizes some of the common terms used in AI and AM). This geometric information, obtained by computed tomography, laser scanning, structured-light scanning, stereovision scanning, and optical coherence tomography [82,83,84], is then used by the AI algorithms to determine the best toolpath design and the stimuli-responsive material distribution, allowing precise and predictable shape control of the 4D-implant so that it can be used in a minimally invasive way and, for example, expand to fill the target space after deployment to the target position or transform over time as the body heals. Intriguingly, as reported by Zhijie Zhu and co-workers [85], in the case where a target surface is not present (e.g., a damaged nerve pathway or an occluded blood vessel), and therefore there may be issues related to the substrate for the printing process, AI can use a library of previous scans of the target region of interest and couple it with incomplete anatomy scan data to reconstruct a patient-specific regenerative implant model to be fabricated through 4D printing. 

Such open-loop AI-based approaches can be used for the fabrication of 3D anatomical models or implants that recapitulate perfectly the microscopic anatomical structures of damaged tissues and organs, thus minimizing the manual intervention for printing on complicated geometries and having a positive impact in terms of accuracy of surgery and efficiency of tissue regeneration, as it is possible to obtain a perfect match between the 4D-printed part and the target complex surface. 

**Figure 5:**
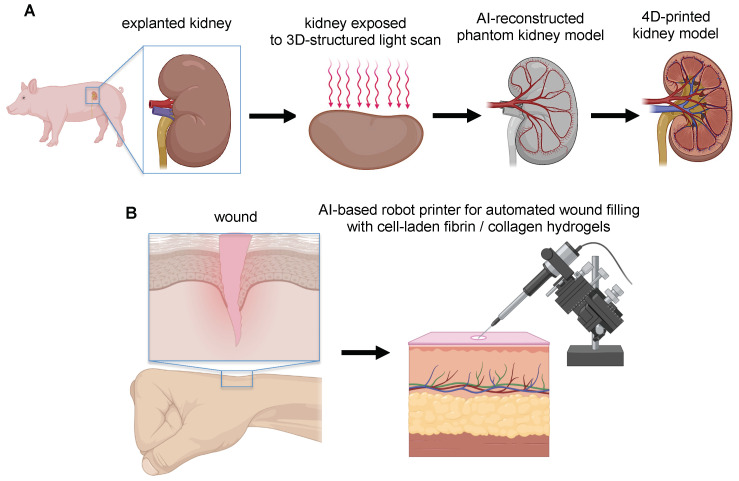
Schematic representation of open-loop AI-based 3D printing approaches. (**A**) The open-loop structured light scan that allows the topographical matching of the geometry of the 3D-printed phantom biomimetic substrate with the anatomy of the porcine kidney, for fabricating microfluidic device conformably to this substrate and subsequently implantable to the surface of the porcine kidney. (**B**) Personalized skin 4D printer to print cell-laden fibrin/collagen hydrogels on wounds in real time thanks to the coupling of computer-vision algorithms and structured-light scanner.

Manjot Singh et al. [86] reported the use of open-loop structured-light scanning techniques that enable the topographical matching of 3D-printed device geometry to porcine kidney anatomy. The structured-light 3D scanner digitally projects visible light patterns onto the target surface and captures the reflected light patterns with cameras. These light patterns captured by the cameras are then used via AI to calculate the 3D shape of the target surface (Figure 5A). This method has been used to scan a porcine kidney for fabricating a microfluidic device directly interfaced with a porcine kidney for organ assessment. Based on the 3D scan of the kidney, the authors 3D-printed, for the first time, a phantom kidney as a biomimetic substrate. Subsequently, the microfluidic device was conformably printed onto this biomimetic substrate and then transferred to the kidney surface. The authors argued that using this open-loop AI-based 3D printing approach bypassed the challenges of direct 3D printing onto a living kidney surface, such as real-time compensation for organ deformation and movement. 

Instead, Mohammed Albanna et al. [87] developed and validated a mobile skin bioprinting system that incorporates a structured-light scanner to extract the 3D contour of a wound region and automatically recognize, through an AI system, the region to be repaired (Figure 5B). Such integrated imaging technology with bioprinting facilitated the precise delivery of dermal cell-laden fibrin/collagen hydrogels (either autologous or allogeneic) into an injured area, replicating the layered skin structure, providing rapid on-site management of extensive wounds. Moreover, this strategy allowed the delivery of the bioink (i.e., cells + hydrogel) to specific locations of the wound based on wound size and topology, thus resulting in the acceleration of wound healing and the formation of normal skin in situ, compared with untreated samples.

Furthermore, as discussed in Section 2.3, shape-shifting materials that can take and hold any possible shape, or folding, bending, or twisting following applied stimuli could be useful for the fabrication of wearable medical implants directly 4D printed on the human body to perform clinical diagnoses, aid in wound recovery or tissue regeneration. The AI can ameliorate the design and 4D printing of wearable medical devices using acquired scans of deformable target surfaces, making them compliant to the possible body motions by adopting the properties of the functional responsive materials [88,89]. Again, the involvement of AI for shape-changing and shape-memory 4D printing materials is considered an open-loop approach since the AI algorithms are used during the design phase rather than the printing process. This approach will certainly improve the robustness and durability of wearable devices because, on the one hand, materials able to change shape will be used according to the local characteristics of the target tissue or joint movements, and on the other hand, to satisfy the specific needs of each patient.

## 4. Closed-Loop AI for 3D Printing

The closed-loop AI printing integrates the detection of changes in the 4D printing environment (e.g., printing defects, movement or deformation of the target surface, material flow, printing speed, nozzle height, printing temperature), thus adapting the AM process in real-time [90] (see Table 1). Sensory data is processed using AI tools to recognize target surface or print defects, while a feedback control system adjusts the toolpath in real-time to compensate for the target movement, printer calibration errors, and material flow, thus (1) ensuring the 4D printing process, (2) improving printing quality, and (3) enabling in situ printing on moving targets via online tracking.

This strategy can empower 4D printing, providing new possibilities for developing not only stand-alone wearable devices based on responsive materials printed on moving targets [16], but may also be used as innovative biomedical technology for autonomous surgeries, laparoscopy, and endoscopy [91], as well as for the fabrication of 4D printed soft robots with embedded sensors (i.e., strain sensors, tactile sensors, magnetic field sensors, flow sensors, and biosensors) [92].

**Table 1:** Definition of common terms used in AM and AI.

Common Terms Used in Additive Manufacturing and Artificial Intelligence	REFs
**3D printing:** three-dimensional (3D) printing is an additive manufacturing process in which a physical object is created from a computer-aided design (CAD) model by printing the model on a pre-computed layer-by-layer toolpath. This process is fully deterministic and, therefore, is ideal for printing on planar surfaces that are stationary relative to the coordinate system of the printer (namely, ex situ 3D printing). To date, there are several 3D printing methods that include the following: fused deposition modeling (FDM), selective laser sintering (SLS), stereolithography (SLA), and direct ink writing (DIW).	[1,18,24,28,40]
**4D printing:** four-dimensional (4D) printing uses the same techniques of 3D printing through computer-programmed deposition of material in successive layers to create a 3D object. However, in 4D printing, the resulting 3D object is able to change shape, structure, or function directly off the print bed in response to external stimulus, with the fourth dimension being the time-dependent shape change after the printing. It is therefore a type of programmable 3D printer, wherein after the fabrication process, the printed material reacts with parameters within the environment (humidity, temperature, mechanical force, pH, etc.) and changes its form accordingly.	[2,4,47,49,50,59,80]
**Artificial Intelligence:** artificial intelligence (AI) leverages computers and machines to mimic the problem-solving and decision-making capabilities of the human mind. Although a number of definitions of AI have surfaced over the last few decades, the most used is that of John McCarthy: “it is the science and engineering of making intelligent machines, especially intelligent computer programs. It is related to the similar task of using computers to understand human intelligence, but AI does not have to confine itself to methods that are biologically observable”.	[56,58,87]
**Machine Learning:** machine learning (ML) is a branch of AI and computer science, which focuses on the use of data and algorithms to imitate the way those humans learn, gradually improving its accuracy. ML involves the development and deployment of algorithms that, rather than being programmed to assign certain outputs in response to specific inputs from the environment, analyze data and their properties, and determine the action by using statistical tools. Usually, ML algorithms can be broadly classified into the following five categories: supervised learning, unsupervised learning, semi-supervised learning, reinforcement learning and federated learning.	[56,57,87]
**Open-Loop AI printing:** open-loop AI leverages pre-acquired sensory data (such as laser scanning and 3D tomography reconstructions) to obtain precise target geometry in various forms of 3D representations such as meshes and voxels. Then this geometry is calibrated with respect to the printing platform, thus enabling the generation of a toolpath on complex surfaces (i.e., organs or tissues). Based on this morphing path, open-loop AI can design the distribution of shape-morphing materials (whereby the morphing can be induced by mechanical load, change of temperature or pH, swelling) within the 3D-printed model to achieve improved compliance to a dynamically varying target surface. The AI-related computation occurs prior to the printing process.	[87,90]
**Closed-Loop AI printing:** closed-loop AI printing integrates sensing as part of the printing process. The sensory data are processed in real time using AI tools to recognize the surface of the target. A feedback-control system adjusts the toolpath in real time to compensate the target motion, environmental disturbance, and calibration errors, thus ensuring the 3D printing procedures.	[87,90,92]

For instance, soft organs and tissues, such as the lung, heart, and skin, undergo continuous deformations; therefore, they cannot be completely immobilized on a surface for in situ 3D printing, thus requiring closed-loop AI tools with online updates for adapting the printing toolpath in real-time. The closed-loop AI inkjet printing on a moving lung was recently demonstrated by Zhijie Zhu and colleagues [93]. This closed-loop strategy enables the estimation of motion and deformation of the target lung surface to adapt the in situ printing toolpath in real-time. With this printing system, the authors demonstrate the possibility of printing a hydrogel-based sensor on a porcine lung under respiration-induced deformation with a tracking error of 0.65 mm. In a similar manner, the authors also developed a real-time closed-loop system that tracked the motion of a human hand to perform in situ 3D printing of electronic tattoos directly on the skin [16].

Such adaptive closed-loop 3D printing approaches could be used in the near future to enhance robot-assisted medical treatments with AM capabilities, enabling autonomous and direct printing of stimuli-responsive materials on and inside the human body. 

**Figure 6:**
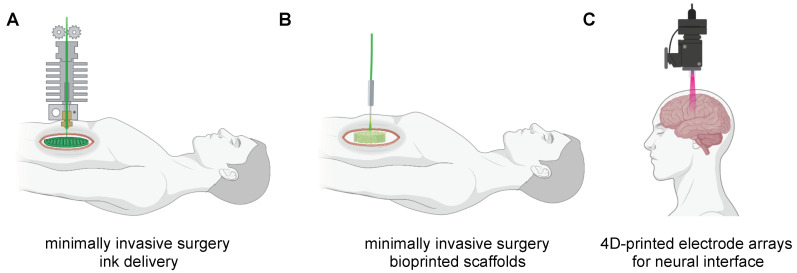
Schematic representation of closed-loop AI-based 4D printing approaches for autonomous minimally invasive surgery (MIS). (**A**) 4D inks delivery with biological and mechanical features mimicking the human body tissues and organs. (**B**) In situ bioprinting scaffolds with engineered cells to repair damaged tissues/organs. (**C**) Surgical robot-assisted implantation of 4D printed electrode arrays for neural interface.

Hence, the concept of closed-loop AI-based 3D printing could also be integrated into surgical robots for minimally invasive surgery (MIS), which constantly measures the exact size of the defect thanks to the continuum of endoscopic imaging, to better performed either in situ printing procedures and “smarter surgery” to reduce deaths or injuries due to medical error. Different researchers [94,95,96,97,98,99,100] have envisioned the possibility that MIS robotic arms could carry printer nozzles, controlled by computers, scanners, and AI tools to (1) deliver inks with suitable mechanical, chemical and biological functions directly to the human body during the MIS procedures (Figure 6A); (2) bioprinting scaffolds with engineered cells to repair or replace damaged tissues/organs (Figure 6B); (3) implant 4D printed electrode arrays for neural interface (Figure 6C). Despite such approaches are exceptional, there are only a small number of surgical robot-assisted devices that can be integrated with 4D printing and AI tools, as well as such technology due to their infancy significantly suffers from low sensing, slow printing speed, and lower resolution Given the rapid growth of such technologies, we expect these gaps to be filled soon.

## 5. 5D Printing: A New Route of AI and AM

Looking ahead, Milazzo and Libonati recently reported an interesting perspective on AI-empowered 3D and 4D printing approaches [55]. The authors expect that in the future, the synergistic contribution of AI and its subset ML will give life to “5D printing”, in which AI will assume the fifth dimension. The collaborative and integrated approach between AI and AM, which leverages stimuli-responsive materials, will ensure novel opportunities not only for the intelligent fabrication of components with multiple functions, but also for the fabrication of ecofriendly and biocompatible living materials (i.e., fisheries chitin, nano-cellulose, and silk fibroin, to name a few) [101]. 

Moreover, in the biomedical fields 5D printing could be used for developing protective bandages or bio-patches that may detect signs of infection or disease, as well as for design and triggering selective sets of features, optimal for specific functions (e.g., drug delivery based on shape mutation [102,103], optoelectronic properties triggered by changeable textures [104,105], or activate new properties, currently not found in nature.

Although 5D printing has the potential to revolutionize the field of AM and the production of smart materials that can be printed in situ, many challenges still need to be addressed to achieve the complete versatility of this approach.

In our opinion, the most relevant issue concerns the full scalability of the process, as the AI tools that should control the whole process are not yet integrated into the 3D and 4D printers, nor in the ability to detect, adapt and predict the materials to be used. For the latter, AI-based optimizations will be continually refined to achieve high levels of accuracy in predicting the behavior of a material or device, but the main bottleneck is based on the current limitations possessed by AM technologies in terms of repeatability, resolution, and accuracy. Indeed, the AI tools are isolated computing blocks that take sensory data as input and produce processed measurements and control commands as output, but they still lack an interactive interface between the 3D/4D printer and the user.

When AI interfaces are integrated into the 3D printing process, AI will drive the entire end-to-end process, from computational design to target-specific in situ printing based on a large database of human-printer interactions.

All the aforementioned will inevitably lead to drawbacks in terms of costs that will have to be taken into consideration and addressed in the near future, given that a cornerstone of 3D and 4D printing technologies is based on the design and prototyping of complex architectures, reducing costs compared to conventional processes.

## 6. Regulatory Standpoint for AI

It becomes clear that AI and ML have caught the world’s attention as leading technologies that can shape the future of 3D- and 4D printing for personalized medicine, regenerative medicine, tissue engineering, and robot-assisted medical treatments. Accordingly, the abovementioned distinctive capabilities afforded by AI tools have introduced new regulatory challenges that must be considered and carefully addressed in light of the fact that either AI or ML tools could be applied with reference to high-risk activities (e.g., medical implants, drug delivery, replacing damaged tissues/organs, etc.) that could cause serious damage to final users. In other words, close attention will have to be paid to the regulatory framework to ensure the safe technological transfer of AI from the “proof of concept” to the application in the real world. 

Indeed, in April 2021, the European Commission released a regulation proposal, called the AI Act, aimed at the safe and efficient development, implementation, and use of AI in different fields of applications [106]. A first constraint to be addressed is the univocal definition of AI, which will determine the scope of the regulation, as a narrow definition would leave some types of AI systems out of the scope; however, too broad a definition risks wiping out the common algorithmic systems that do not produce the types of risk or harm that AI regulation focuses on [107]. Therefore, the definition that will be adopted in the AI Act will likely become a benchmark for other AI regulations in other countries outside of Europe, thus helping to build a global consensus. 

Second, the AI Act regulation proposal emphasizes regulatory burdens when an AI system presents high risks to fundamental rights and end-user safety [107,108]. The AI Act classifies risk into the following four levels: unacceptable risk, limited risk, minimal risk, and high risk. For the high-risk AI-based systems, they will be subject to the following strict obligations before they can be placed on the market: adequate risk assessment and mitigation systems; high quality of the data sets that feed the system to minimize risks and discriminatory outcomes; recording of activities to ensure the traceability of results; detailed documentation providing all necessary information about the system and its purpose for the authorities to assess compliance; clear and adequate information to the user; adequate human supervision measures to minimize the risk; the high level of robustness, safety and precision. Globally, many governments support that AI regulation should be risk-based. In 2021, the FCAI report strengthening international cooperation on AI found that most government participants explicitly endorse a risk-based approach to AI regulation. For instance, the United States Office of Management and Budget’s Guidance for Regulation of Artificial Intelligence Applications already includes “risk assessment and management” as one of its principles. However, moving from a high-level commitment to high-risk assessment to its application will reveal different approaches that, if not addressed, threaten to lead to different localized approaches to assessing AI risk and risk management that can create costs for AI development and use.

In the U.S.A., the National Institute for Standards and Technology (NIST) is developing an AI Risk Management Framework (AI-RMF) that could facilitate alignment on approaches to identifying and assessing risk. That said, there are already emerging differences in the U.S. and EU approaches to risk assessments for AI [109]. For example, the AI Act’s division of AI systems into four risk categories may not be reflected in the U.S. approach. In addition, the U.S. has already emphasized that any assessment of AI risk needs to take into account the extent AI systems improve on existing risks, whereas the EU AI Act does not currently explicitly address this issue. Furthermore, the proposed requirements for high-risk AI cannot always mitigate the damage to health, safety, and fundamental rights that these practices entail. Hence, the need to introduce a complaint or redress mechanism for people who suffer damage from AI systems. The European Economic and Social Committee (EESC) fills this gap by asking the Commission to implement such a system so that Europeans have the right to challenge decisions made exclusively by an algorithm. More generally, according to the EESC, the AI Act does not specify that the promise of AI lies in enhancing decision-making and human intelligence. It works on the premise that once the requirements for medium- and high-risk AI are met, AI can largely replace human decision-making.

## 7. Conclusions

In the past decade, stimuli-responsive materials have begun to attract attention thanks to their ability to perform living-like functions such as self-adaptability, self-repair, shapeshifting, shape-memory, and self-assembly in response to chemical, physical and biological cues. The 4D printing technology led to a breakthrough in materials science, as by preprogramming smart materials, the 4D printing is able to manufacture structures that dynamically respond to external stimuli, adapting their features and functions for specific applications, exerting positive effects on various biomedical applications. However, as is pointed out in this review, despite the fact that progress has already been made in this field, many limitations and challenges remain to be overcome. 

First, 4D printing technology is in its infancy, and its printing of stimuli-responsive materials is still in its exploration state. Second, another major drawback lies in the nature of either 3D- or 4D printing, where designed structures are typically manufactured on a planar and flattened substrate and then transferred to the target non-planar surfaces, such as those of the human body. Therefore, the printing procedure is fully deterministic, leading to a possible mismatch between the printed implants and the target surfaces of the human body. Third, the challenges and prospects for the progress of 4D printing technology lie in the ability to in situ print smart materials in order to allow dynamic and complex actions such as self-adaptability, self-repair, shape-changing, and self-assembly, on organs and tissues that undergo continuous deformations and motions, so far not always easy to make. 

In this context, AI could push forward these technologies by expanding their applicability, thus paving the way to the concept of 5D printing, in which AI will assume the fifth dimension. Indeed, we highlighted that the open-loop AI approach can be useful for reconstructing a patient-specific regenerative implant model to be fabricated through 4D printing, while the closed-loop approach can be a valuable tool to integrate into surgical robots for minimally invasive surgery (MIS) in order to better perform in situ printing procedures to reduce deaths or injuries due to medical errors, as well as for bioprinting of smart scaffolds with engineered cells to repair damaged tissues and organs. In view of this, the role of AI will be pivotal in accessing and analyzing data not only from/for the printing process but also from experimental datasets that will improve the learning process for real-case scenarios. Therefore, we are confident that in the near future, groundbreaking research in 5D printing will naturally fill the aforementioned limitations, as well as the risk assessment and management due to the use of AI. In our opinion, we have only scratched the surface of the development possibilities of the collaborative approach between AI and AM technology, and we foresee a number of great opportunities for future research in the biomedical field and in industry 4.0, in particular for the smart manufacturing, the fabrication of ecofriendly and biocompatible living materials, in situ printing on moving targets, and “smarter surgery”. However, it must be noted that AI and ML in the AM and biomedical fields are high-risk technological solutions that could cause serious damage to end-users. Therefore, it is becoming increasingly necessary to have a clear regulatory framework that takes into account the risk management of these technologies to preserve the fundamental rights and safety of end-users and developers of these systems.

## Data Availability

Not applicable.
